# Supplementary material for: Saccharomyces boulardii (CNCM I-745) improves intestinal damage in sepsis by remodeling intestinal flora structure
Source: Front Cell Infect Microbiol. 2025 Jul 31;15:1602792. doi: 10.3389/fcimb.2025.1602792 (PMC12350329; doi:10.3389/fcimb.2025.1602792)
Supplement: Supplementary file 1 [file DataSheet1.zip › summary/summary/8_Advanced_analyse/2_PICRUSt2_result/METACYC/Group.DW4_vs_BW4/PICRUSt2_METACYC_Group.DW4_vs_BW4_diff.pdf]

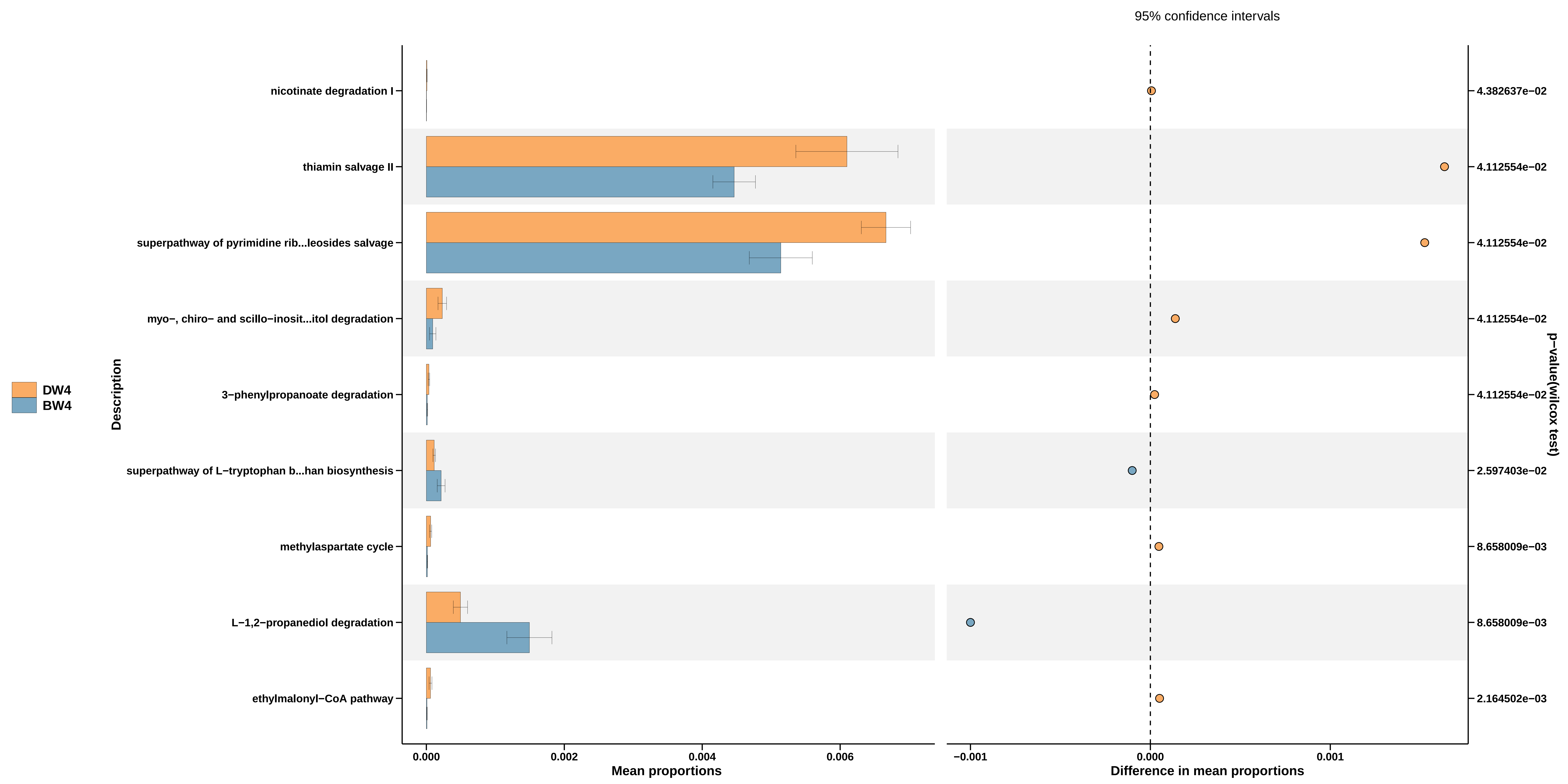

95% confidence intervals
nicotinate degradation I
thiamin salvage II
superpathway of pyrimidine rib...leosides salvage
myo−, chiro− and scillo−inosit...itol degradation
3−phenylpropanoate degradation
superpathway of L−tryptophan b...han biosynthesis
methylaspartate cycle
L−1,2−propanediol degradation
ethylmalonyl−CoA pathway
Description
DW4
BW4
0.000
0.002
0.004
0.006
Mean proportions
−0.001
0.000
0.001
Difference in mean proportions
4.382637e−02
4.112554e−02
4.112554e−02
4.112554e−02
4.112554e−02
2.597403e−02
8.658009e−03
8.658009e−03
2.164502e−03
p−value(wilcox test)
